# Commentary: Meta-Analysis and Structural Dynamics of the Emergence of Genetic Variants of SARS-CoV-2

**DOI:** 10.3389/fmed.2021.747109

**Published:** 2021-09-23

**Authors:** Sara Haddadi, Mehdi Mirsaeidi

**Affiliations:** ^1^Department of Medicine, Division of Pulmonary, Critical Care, Sleep and Allergy, University of Miami Miller School of Medicine, Miami, FL, United States; ^2^Division of Pulmonary, Critical Care, and Sleep Medicine, College of Medicine, University of Florida, Jacksonville, FL, United States

**Keywords:** COVID-19, India, pandemic, SARS-CoV-2, vaccination, variants

## Introduction

While new variants of severe acute respiratory syndrome coronavirus 2 (SARS-CoV-2) have been spreading rapidly throughout the globe, genetic modifications, and impact of emerging variants on neutralizing antibody vaccines was discussed by Castonguay et al. (1) Considering the globe yet under vaccinated and the high infectivity of Delta variant which was first detected in India, we identified factors associated with the second wave of COVID-19 in India and proposed measures to overcome the pandemic.

After more than a year since the beginning of COVID-19 pandemic wearing masks has become a social norm in many countries. Following the increase in administration of COVID-19 vaccines in the US, CDC announced a mask off policy for fully vaccinated individuals (2). Some scientists have been opposing this change in the policies due to the higher infectivity and unpredictability of new variants. Variants of concern which contain spike protein mutations may escape vaccines' neutralizing antibodies (3). Pfizer company recently announced that they recommend third dose of the vaccine implementation while the US Department of health and human services declared fully vaccinated Americans do not need a booster dose at this time (4). The debate about the vaccination doses is increasing in the light of scientific discoveries about SARS-CoV-2 new variants. Meanwhile, some countries are still struggling to have one or the second vaccine doses. Preventive policies are essential in countries with a lower vaccinated population. Moreover, some studies suggest the necessity of an extra vaccine dose in immunocompromised patients (5).

Mix and match strategies for booster vaccines such as combining the Oxford-AstraZeneca and Pfizer-BioNTech doses have been tested and suggested due to safety concerns and unpredictable supplies (6). Further evaluations are warranted for early detection of emerging SARS-CoV-2 variants that may escape the vaccines' neutralizing antibodies. Other preventive measures should remain unchanged. We aimed to discuss factors that led to a second wave COVID-19 in India, a vaccine producing country, and emergence of Delta variant.

## Delta Variant in India

India's second wave of COVID-19, and the subsequent mitigating factors during the vaccination era is an important example of the emerging viral variants effects on pandemic in populated areas as India and the requirement of rapid global response. Experience from prior pandemics such as Influenza in 1918 and 2009 showed that not only two but subsequent waves of infection is expected (7). Mandal et al have evaluated the plausibility of a third wave of COVID-19 in India. They found two potential mechanisms for a third wave including “a new variant that is more transmissible and at the same time capable of escaping prior immunity,” and release of lockdowns that are highly effective in limiting transmission. It is unlikely that a new highly transmissible variant would exceed the threshold (R04 > .5) to cause a third wave by itself. As a result, keeping up with preventive measures and rapid increase of vaccination play an important role in preventing the future waves (8). B.1.617 lineages of SARS-CoV-2 was detected as a dominant variant in India and B.1.617.2 also called Delta variant that was first detected in December 2020 became the most frequently reported variant from mid-April 2021 (9).

The risk of new viral mutations increases in populations with a high rate of transmission. India was hit by more than 300,000 new cases of COVID-19 per day during the last days of April 2021 and the numbers were rising in May 2021. On May 4th, 2021, WHO declared the highest level of COVID-19 for the second consecutive week since the beginning of the pandemic with over 5.7 million new weekly cases (10). On May 7th, 2021, a report of 414,188 new cases of the infection in 24 h, reached to a global record. On the same day, WHO authorized China's Sinopharm vaccine for emergency use following nine consecutive weeks of rise in global COVID-19 cases (11).

## COVID-19 Crisis Associative Factors in India

As the pandemic started, India closed its international borders and imposed a lockdown quick enough that WHO praised it as “tough and timely”. However, health inequalities, economic and social disparities caused unique challenges in India during the pandemic. Although some of the country's states were enforcing rigorous laws for testing and monitoring such as control of social distancing via drones during the lockdown, overall testing rates have been low. Apart from the high capacity and lack of operational feasibilities, fear, stigma, and blame were among the misinformation threats that delayed COVID-19 testing (12).

In late March 2021 when the results from serological surveys and India's main computer model predicted the rate of the spread of the disease, the Indian government called it “endgame” of the pandemic. By that time restaurants, shopping centers and theaters had been reopening in the country. There has been calls by public health experts for accurate data and preventive measures which were overlooked (13).

On May 7, 2021, CDC updated the category of SARS-CoV-2 transmission mode to “inhalation of virus” and the virus being an airborne threat (14). This warrants further attention to social distancing in crowded populations.

Although India has the world's biggest vaccine manufacturers, the vaccines availability did not suffice the demands during the crisis (15). Closing borders started soon after the COVID-19 crisis in India, however, the country's neighbors such as Nepal reported a rise in their new COVID-19 cases during May 2021. This viral spread can result in a worldwide spiral. We strongly recommend 3rd dose of vaccine everywhere that has access to the vaccines. Furthermore, due to the lower efficacy of current vaccines against Delta variant we urge production of a vaccine that would be specifically developed for Delta variant.

## Discussion

The India's catastrophic humanitarian situation calls for close attention to the policies that may result in reopening too quickly even after the pandemic is waning following vaccination in many parts of the world. New viral variants may escape from neutralizing antibodies and could emerge rapidly due to the magnitude of infected patients. The world should understand the serious risk of losing battle against COVID-19 if under vaccinated areas remain uncontrolled for the next coming months.

## Author Contributions

SH and MM: conceptualization, methodology, investigation, and writing—review & editing. MM: validation and supervision. SH: writing—original draft preparation. Both authors contributed to the article and approved the submitted version.

## Conflict of Interest

The authors declare that the research was conducted in the absence of any commercial or financial relationships that could be construed as a potential conflict of interest.

## Publisher's Note

All claims expressed in this article are solely those of the authors and do not necessarily represent those of their affiliated organizations, or those of the publisher, the editors and the reviewers. Any product that may be evaluated in this article, or claim that may be made by its manufacturer, is not guaranteed or endorsed by the publisher.

## Abbreviations

CDC: Centers for Disease Control and Prevention
COVID-19: Coronavirus Disease 2019
SARS-COV-2: severe acute respiratory syndrome coronavirus 2
WHO: World Health Organization.
